# Non-Standardized Methods of Assessing Tibial Loads During Different Gait Speeds Obscures Load-Management Recommendations in Healthy Adults

**DOI:** 10.3390/s26144401

**Published:** 2026-07-10

**Authors:** Jack D. Hart, Eric J. Drinkwater, Elizabeth J. Bradshaw

**Affiliations:** 1 Centre for Sport Research, Institute for Physical Activity and Nutrition, School of Exercise and Nutrition Sciences, Deakin University, Burwood, VIC 3125, Australiaeric.drinkwater@deakin.edu.au (E.J.D.); 2 Sport Performance Research Institute New Zealand, Auckland University of Technology, Auckland 1142, New Zealand

**Keywords:** biomechanics, gait analysis, wearable sensors, inertial measurement units (IMUs), rehabilitation

## Abstract

This scoping review aimed to investigate (i) the methods used to measure tibial load during human gait, and (ii) the effect of gait mode and velocity on tibial load in healthy adults. This review followed the guidelines of the Preferred Reporting Items for Systematic Reviews and Meta-Analyses Extension for Scoping Reviews (PRISMA-ScR). Two databases (EMBASE, MEDLINE) were searched, revealing twelve studies that met the inclusion criteria from the previous 24 years. Tibia load was measured using indirect (in vivo strain gauges) and direct (accelerometers, inertial measurement units [IMUs]) sensor methods, and three-dimensional motion analysis systems. A range of surfaces were used, including treadmill (motorized, curved non-motorized) and overground conditions. Velocity was a key determinant of tibia load, with surface type and gait modifications further influencing the loads. The non-standardized measurement methods resulted in varied tibial load results, particularly from varied anatomical positions used in indirect sensor methods. The findings suggest that, based on the current literature, prescribing load management recommendations for healthy adults is not currently possible, given the variability in results. Future research should aim to develop standardized measurement protocols to improve injury risk reduction strategies and to inform rehabilitation programs to support individuals in resuming participation in gait-related activities.

## 1. Introduction

The health and wellbeing benefits of exercise are widely accepted, and walking and running are popular modes of exercise for many adults [1]. In adults, walking in particular is associated with a reduction in cardiovascular disease, hypertension, type 2 diabetes, osteoporosis, and various cancers, as well as anxiety, depression, and cognitive decline in older adults [2]. Similarly, running promotes many of the same physiological health benefits as walking [3] when matched for energy expenditure. Running achieves the same energy expenditure as walking in a shorter time, but also places more load on the body, which has implications for its benefits and risks.

*Load* is defined as ‘mechanical stress applied to bone’ [4] (p. 154) and is a critical factor in understanding bone health. Load can result in micro-deformation and/or bending, which results in bone *strain*. Musculoskeletal load and stress during human gait results from a combination of external ground reaction forces (GRFs) when the foot contacts the ground, and the internal forces and torques (i.e., inertial moments) through and between the segments (e.g., joint reaction forces) [4]. The importance of musculoskeletal loads on the human body extends to physical adaptations, such as in the maintenance and/or increase in bone mineral density [5]. Walking is a viable exercise option for bone health, particularly for older adults, and has the benefit of being easy to accommodate into one’s lifestyle. Running, however, leads to stronger outcomes to bone health (density and strength), especially in younger adults who can more often safely engage in higher intensity exercise [5]. Although running provides a solid foundation for positive adaptations and reduced associated health risks, higher training volumes can also be associated with injury [6]. Common injuries seen in runners have a primary mechanism of overuse (repetitive stress) [7] including tibial stress syndrome (colloquially known as shin splints) and tibial stress fractures [6]. Understanding tibial loads and the factors that influence such loads during human gait is important for assessing injury risk, as tibial injuries are understood to be influenced by mechanical load and stress [6].

Two movement phases comprise human gait as a cyclical action: the stance phase, where one or both limbs contact the ground, and the swing phase, where one or both limbs are rotating in the air [8]. The tibia is loaded during the stance phase where the external GRF is imparted through the plantar surface of the foot, resulting in internal loading of the segments and joints of the lower limb and trunk [9]. The axial (longitudinal) forces reach up to 4.7 × body weight (BW) along the tibia during the late stance [10]. There are two peaks of tibial loading during walking gait, which occur during early and late stance [10]. Axial loading has been established as being greatest in the distal tibia, whereas the proximal tibia was reported to have the greater bending torques [10,11]. Axial loading patterns such as peak tibial acceleration has been correlated with the external vertical GRF loading rate for running [12]. However, the validity of measuring GRFs to estimate tibial force has been questioned [13]. One study [14] reported that GRF metrics do not strongly correlate with tibial loading, and that GRF metrics should not be assumed to reflect internal tibial forces or injury risk. The authors argued that the majority of tibial bone load was associated with muscle forces (gastrocnemius and soleus), as opposed to simply the external GRFs. Additionally, the authors contended that peaks in GRF/time curves did not coincide with peaks in tibial load/time curves, and that muscle forces can increase without a corresponding increase in GRF [14].

Methods of measuring tibial loads include direct and indirect methods of measurement. Zandernberg et al. [15] classified indirect methods as including wearable accelerometers, as they are limited to measuring external loads such as GRFs and tibial accelerations. They stated that direct measures can only be obtained in vivo after invasive surgery to embed micro-devices such as strain gauges, but can then measure both internal and external forces through the tibia. To examine the relationship between direct and indirect methods of measuring tibial loads, the relationship between peak tibial acceleration and estimated maximum tibial compression forces was examined during running. Results indicated that there was a negligible (r < 0.1; Ref. [16]) correlation between maximum tibial compression forces and tibial accelerations when running at a single speed [15]. It suggests that peak tibial acceleration (PTA) should be reconsidered as an indicator for tibial bone loading. Despite these concerns, two studies have examined the effect of running gait retraining to reduce tibial load [9,17]. Real-time feedback resulted in moderate reductions in tibial loads, whereas other more common interventions such as increasing cadence or switching to a forefoot striking technique does not significantly reduce tibial loads and therefore may not have a strong impact on reducing tibial stress injuries.

### 1.1. Population, Concept, and Context (PCC) Statement

This scoping review will examine literature that reports original scientific research that has utilized objective methods to measure tibial loads (Concept) during human gait of adults (Population). An improved understanding of tibial loads when walking or running at different speeds, and on different common surfaces, will give insight into any existing consensus involving practices for measuring and monitoring this biomechanical metric in exercise prescription and rehabilitation (Context).

### 1.2. Aim

The scoping review will examine the effect of (i) different speeds and (ii) surfaces on tibia loading during human gait in healthy adults. Included in this scoping review will be the identification of the prevalent indirect (non-invasive) and direct (in vivo) methods of assessing tibial load during human gait, to ascertain whether the lack of standardization of tibial-loading assessment methods affects overall knowledge of tibial loads during human gait.

## 2. Materials and Methods

### 2.1. Data Sources and Search Strategy

This scoping review followed the guidelines of the Preferred Reporting Items for Systematic Reviews and Meta-Analyses Extension for Scoping Reviews (PRISMA-ScR) checklist [18] and was managed using Covidence online software [19]. A two-database search was completed based on tibial loading at difference speeds of human gait on the EMBASE and MEDLINE databases [20] published between January 2000 and August 2024. Key words used for the search included:

1. Tibia OR lower leg*.
2. Stress OR compress OR tensile* OR shear* OR accel* OR load* OR strain* OR shock*.
3. Walk* OR jog* OR run* OR sprint* OR gait* OR locomotion OR stride*.
4. English AND adult AND humans AND (2000–2024).

### 2.2. Study Selection

Articles identified through the database were title- and abstract-screened, based on the inclusion and exclusion criteria and to remove any duplicates by two authors (JDH and EJB). Full-text screening was conducted to assess eligibility by the same two authors on the *a priori* inclusion and exclusion criteria. Any disagreements were resolved by consensus. Secondary searches were conducted by scrutinizing the reference lists of each full text that met the inclusion criteria.

#### Inclusion/Exclusion Criteria

Inclusion criteria for the selected studies were:

1. Inclusion of a measurement of tibial acceleration, force, or load.
2. A measurement of what activity was being done and/or the speed it was completed at.
3. Full text article.

Exclusion criteria for the final selected studies were:

1. Participants were under the age of 18 years.
2. The study was not reported in English.
3. A measurement of the tibia was absent.
4. The participants had current injuries or pathology.

### 2.3. Data Extraction and Analysis

No formal quality assessment or risk of bias analysis of the included studies was undertaken prior to data extraction and analysis, as the purpose of this scoping review was to map existing evidence on this topic [21]. Data from included studies were extracted into table format. Extracted data from each study included (1) the authors and year the study was published, (2) participant details, sample size, and inclusion criteria, (3) the gait mode, speed, and surface examined in the study, (4) the footwear used in the study, (5) the tibial-load measurement methods, including the sensor and/or reflective marker placement protocol, (6) the study purpose, (7) the research design, (8) the study outcomes, (9) the strengths and (10) the limitations of the study. All participant height and body-mass reports were converted to meters and kilograms, where required, and reported as an average and standard deviation (SD), to enable consistent reporting and 95% confidence intervals were converted to SD ($\mathrm{SD}=\surd n\;\times \;\frac{\mathrm{Upper}\;\mathrm{Limit}\;-\;\mathrm{Lower}\;\mathrm{Limit}}{3.92}$) [22]. The research design was classified based on the definitions of Slater and Hasson [23].

The methods of each study were evaluated to identify the data collection setting including the surface the gait task was performed on, the gait mode and speed assessed, the measurement tool used, the placement/position of sensors and/or retroreflective markers, the sampling frequency, and the measures of tibial load obtained. Extracted biomechanical data from each study were quantifiably analysed. Where results were published in figures, WebPlotDigitizer software (version 5.2, Automeris LLC, Pacifica, CA, USA, automeris.io/wpd/) was used for data extraction [24]. Reliability of data extraction was determined from repeat digitization (5 trials) of the first subscale encountered (peak compression in MPa) for eight points using SPSS software (v30, IBM, New York, NY, USA) and demonstrated excellent reliability (α > 0.99, ICC > 0.99). All data extraction was done by one author (EJB) who is experienced in two- and three-dimensional kinematic analyses of biological movement. All numeric data were normalized, where needed, to enable descriptive comparison of findings between studies. Ground reaction-force data were normalized with respect to reports on the participants’ standing body weight (BW), and linear accelerations were normalized with respect to the absolute gravitational constant (g) of 9.81 m/s^2^.

Finally, recurring methodological shortcomings or inconsistencies were identified during the study evaluation and data extraction, and subsequently mapped to provide a checklist for a proposed reporting framework to strengthen future research on this topic.

## 3. Results

### 3.1. Search Results

**Figure 1:**
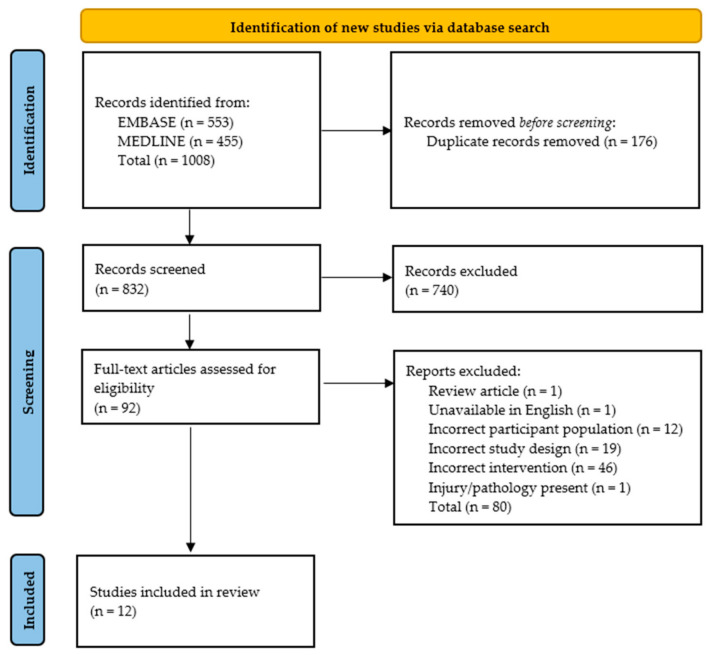
PRISMA-ScR diagram of search results and article screening.

The initial database search yielded 1008 results, of which 176 were duplicates and were removed. Screening of titles and abstracts was then performed, followed by a full text review for potentially selected articles. A total of 820 articles were excluded via the screening process, with a total of 12 studies included in the final review [10,25,26,27,28,29,30,31,32,33,34,35,36]. Results of the search are summarized in Figure 1 and Table 1, Table 2 and Table 3.

### 3.2. Study Characteristics

The studies in this review investigated tibial loads at different speeds of human gait. Most studies used a quasi-experimental research design [26,27,30,33,34,35,36], followed by four cross-sectional design studies [28,29,31,32], and one experimental study design with participants randomly allocated to the intervention or control group [25]. Studies had differing samples sizes, ranging from four participants [32] through to 192 participants [28]. Sample size appeared to be influenced by the study design and measurement methods employed.

### 3.3. Measurement Methods

**Figure 2:**
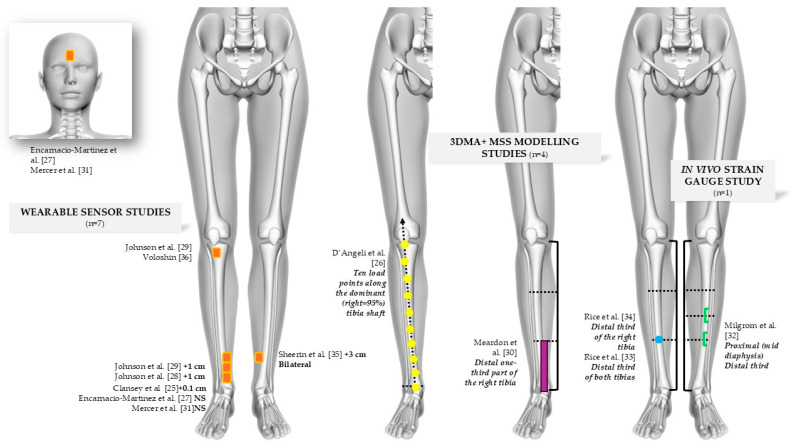
Summary of the biomechanical methods used to measure tibia load [25,26,27,28,29,30,31,32,33,34,35,36], including locations of these measurements using wearable sensors/IMUs (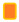), 3DMA/modelling (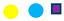), and in vivo strain gauges (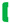) Image credit: kjpargeter on Magnific. URL: https://www.magnific.com/free-photo/3d-female-figure-with-smooth-skin-skeleton_3336312.htm (accessed on 26 June 2026).

Biomechanical measures of musculoskeletal load (Figure 2) included accelerometers and inertial measurement units (IMU; n = 7) [25,27,28,29,31,35,36], three-dimensional motion analysis (3DMA; n = 5) [25,26,30,33,34], and in vivo strain gauges (n = 1) [32]. The region of the tibia commonly measured was the distal endpoint [25,27,28,29,31,35] or distal third [30,32,33,34]. Most wearable sensor (accelerometers and IMUs) studies used absolute positions (+0.1 cm, +1 cm, +3 cm) with reference to the malleoli; however, two studies [27,31] did not state details on the placement of the wearable sensor. One 3DMA study tracked 10 retroreflective markers along the tibia shaft of the dominant limb [26]. Ten studies tested participants on a motorized treadmill [27,28,29,30,31,32,33,34,35,36]; however, one also assessed overground running [28], and one also utilized a curved, non-motorized treadmill [27].

### 3.4. Gait Mode and Speed

Tibial loading, or the amount of mechanical stress exerted on the tibia during human gait, is highly influenced by changes in speed [10,25,26,27,28,29,30,31,32,33,34,35,36]. Speed plays a crucial role in determining the magnitude of the load applied to the tibia during human gait activities like jogging and running. As speed increases, the ground reaction forces that act on the body also increase, which then leads to higher compression and shear forces applied to the tibia [32]. Understanding how these forces vary with speed is essential for injury risk management, and exercise prescription during rehabilitation following injury.

Multiple studies [10,25,26,27,28,29,30,31,32,33,34,35,36] have examined the relationship between human gait speed and tibial loading. This section explores how different speeds of human gait influence tibial loading patterns.

#### 3.4.1. Walking

**Figure 3:**
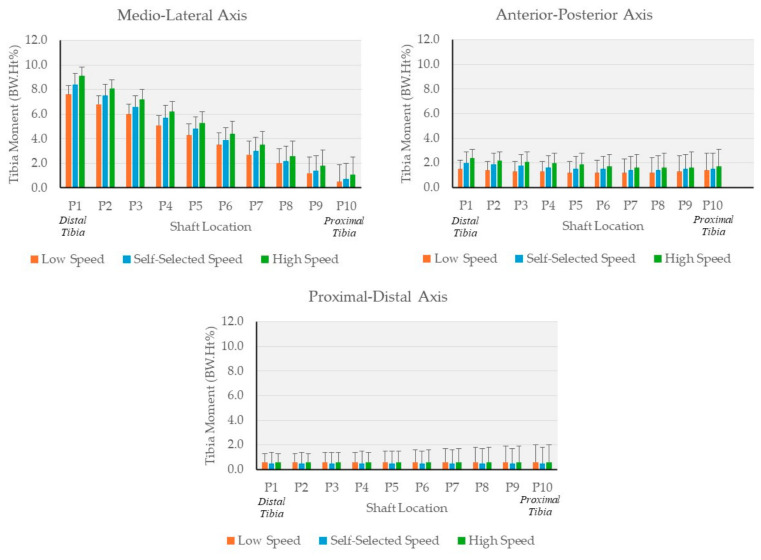
Peak loads on the tibia shaft during low, self-selected, and fast speed walking, as reported in Table 2 of D’Angeli et al. [26]. Peak load was estimated from the moments measured via inverse dynamic methods, for the Medio–Lateral, Anterior–Posterior, and Proximal–Distal axes.

**Figure 4:**
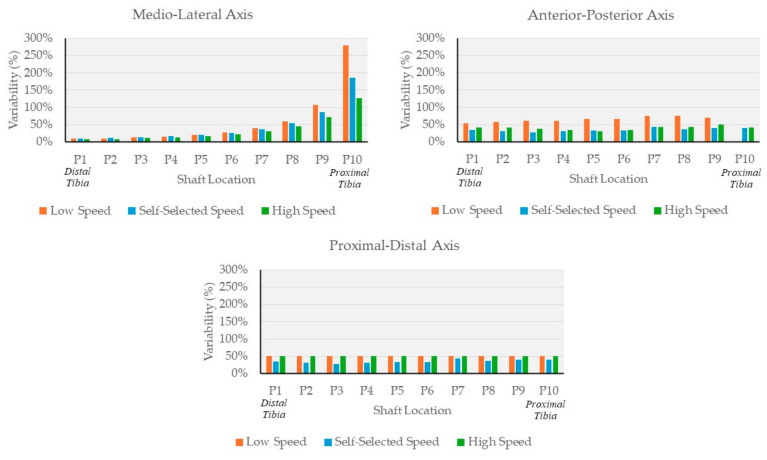
Peak load variability on the tibia shaft during low-, self-selected-, and fast-speed walking, as reported in Table 2 of D’Angeli et al. [26]. Peak load was estimated from the moments measured via inverse dynamic methods, for the Medio–Lateral, Anterior–Posterior, and Proximal–Distal axes.

Four studies [26,30,32,36] measured the tibial loads at different walking speeds. D’Angeli et al. [26] measured the tibia moments using detailed inverse dynamic methods (force platforms synchronized with 3DMA) for 20 healthy adults when walking at their preferred speed and above (faster) and below (slower than) their usual pace. Graphing their tabled results to examine the loads along the tibia shaft provided further insight into their dataset (Figure 3 and Figure 4). It shows that the load patterns were highest in the medio-lateral axis, low in the anterior–posterior axis, and very low in the proximal–distal axis. In the medial–lateral axis these high loads were sustained at the distal-to-middle thirds and were low at the proximal endpoint. The variability observed for these measures was very high throughout the tibia in the anterior–posterior and proximal–distal axes (~40–50%). In the medio–lateral axis the variability was low (~5–15%) in the distal third, but extremely high in the proximal third (~50–280%). The variability in the tibia moments was generally highest when the participants were asked to walk slower than their usual pace and lowest when walking at their usual pace. Finally, their results also indicated that the tibia moments increased with walking speed, specifically in the medio–lateral and anterior–posterior axes. Meardon et al. [30] used 3DMA methods to measure tibial bone stress for 20 healthy adults while walking and running. They reported that tibial stress when walking was half that observed when running. The highest peak compression stress when walking was identified to coincide with the late-stance phase. Milgrom et al. [32] examined the in vivo strain loads at the middle and distal thirds of the tibia of four adult males during walking and running. The in vivo strain gauges were embedded within bone staples and surgically embedded onto the bone. During treadmill walking, the distal tibia sustained approximately 1.95 × higher peak axial compression than the middle tibia. They also observed that treadmill running was associated with peak tensile loads that were 1.94 × measures of walking. Finally, Voloshin et al. [36] measured the effects of walking speed on the peak accelerations at heel strike of the tibial tuberosity (also known as the tibial tubercle; located on the anterior side of the proximal region of the tibia) for 15 healthy adult males using accelerometers. The peak accelerations increased from 2.26 g at walking speed of 0.8 m/s to 5.65 g at walking speed of 1.7 m/s.

#### 3.4.2. Running

Several studies [10,25,27,28,29,30,31,32,33,34,35] measured tibia loads at different speeds of jogging and running. Clansey et al. [25] examined the effects of feedback on tibial shock for running economy in 22 healthy adult males using accelerometers, reporting peak tibial accelerations of 10.67 g for an average running speed of 3.7 m/s. Encarnacion-Martinez et al. [27] also measured peak tibial loads using accelerometers, but for both male and female healthy adults, at running speeds of 2.77 and 3.33 m/s. The tibial loads increased by approximately 16% with the 0.56 m/s (~20%) increase in running speed. Similarly, Sheerin et al. [35] used IMUs to examine peak resultant tibial acceleration of 85 healthy adult runners, showing that these loads increased from 9.8 g when running at 2.7 m/s to 13.5 g when running at 3.7 m/s. Mercer at el. [31] used accelerometers to measure the tibial loads when transitioning from 50 to 100% of maximal running speed for eight healthy male runners, reporting an increase from 6.1 to 10.9 g (~79%). A later study led by Encarncion-Martinez [27] examined variations in tibial shock for a full marathon race of 192 healthy adult runners using IMUs. As the race progressed from the 12th to the 40th kilometre, the average speed of the runners dropped from 3.35 (12.06) to 2.92 m/s (10.51 km/h), with a concurrent reduction in tibial loads from 11.1 to 9.1 g.

#### 3.4.3. Biomechanical Changes with Increased Gait Speeds

Biomechanical changes occur to enable increased gait speed and, as a result, there is increased loads on the tibia. McGrath et al. [49] explored the effects of stride length on lower-limb joint biomechanics at different gait speeds. They reported that increased stride length resulted in greater knee extension during early stance and, similarly, greater knee flexion moments during late stance. The study demonstrated that gait speed had a significant impact on all lower-limb joint moments during the gait cycle, but that stride length was a principal modulator of the lower-limb joint moments, especially for the knee joint. Additionally, Ardestani et al. [50] examined the effects of increased cadence (also known as stride frequency) versus increased stride length as a method of increasing walking speed. They showed that participants who increased their walking speed via increased cadence experienced no significant changes in lower-limb joint moments, whereas participants who increased their walking speed via increasing stride or a combination of increased stride length and cadence experienced significantly greater joint moments.

### 3.5. Human–Surface Interactions

While higher gait speeds increase tibial acceleration loads, the human–surface interaction also influences these loads.

#### 3.5.1. Treadmills

Encarncion-Martinez et al. [27] analysed the effects of running speed (self-selected, 2.77 and 3.33 m/s) on tibial acceleration loads between a motorized and a curved, non-motorized treadmill. The participants’ self-selected running speed was approximately 3% higher on the motorized treadmill (2.82 ± 0.02 m/s) than for the curved, non-motorized treadmill (2.73 ± 0.02 m/s). When the participants ran at their self-selected speed the peak tibial accelerations were 0.49 g (~11%) higher on the motorized treadmill (motorized = 4.78 g, non-motorized = 4.29 g). The same pattern was also observed when they ran at the slower (motorized = 4.97 g, non-motorized = 4.34 g; difference of 0.63 g = ~15%) and faster (motorized = 5.84 g, non-motorized = 5.06 g; difference of 0.78 g = ~15%) set speeds. The tibial accelerations were higher when the runners were asked to run at a non-preferred speed.

#### 3.5.2. Overground Versus Treadmills

Johnson et al. [28] used accelerometers to examine tibial loads when running on a motorized treadmill at 4.06 m/s and overground during a marathon race, starting at 3.35 m/s, and gradually reducing to 3.21 and then 2.92 m/s. The peak tibial accelerations were 11.1 g when running overground at 3.35 m/s, 10.3 g at 3.21 m/s, and 9.1 g at 2.92 m/s. All overground running speeds were associated with higher tibial loads than measures of the treadmill running (8.9 g), despite the lower running speeds observed.

## 4. Discussion

This section discusses potential reasons for varied results from the analysed studies, as well as strengths and limitations of measurement techniques and how they affect the data.

### 4.1. Mechanical Changes Influencing Tibial Accelerations with Surface and Speed

The surface used to measure the effects of gait speed on tibial loads appears to have a significant effect on the results. Encarnacion-Martinez et al. [27] reported greater tibial loads across all speeds when running on a motorized treadmill in comparison to a non-motorized treadmill. Johnson et al. [28] indicated that for most speeds the tibial loads when running overground were greater than when running on the motorized treadmill. Both these studies [27,28] used accelerometers to measure the tibial loads. These findings are likely to be due to changes to gait biomechanics.

Riley et al. [51] observed differences between motorized treadmill and overground running when cadence increased. During treadmill running, stride length shortened, whereas during overground running, stride length increased, as did the ground reaction forces (GRF). The longer strides and higher GRFs when running overground appears to result in the higher tibial acceleration loads observed. Further, Lee and Hilder [52] identified greater knee extensor moments during overground walking, but greater knee flexor moments when treadmill walking. Peak vertical GRFs were the same when walking overground or on the motorized treadmill; however, the horizontal (anterior–posterior) GRF was higher at heel strike during overground walking, which is also likely associated with greater tibial acceleration loads.

### 4.2. Measurement Methods of Tibial Load

This section examines the different measurement techniques used to measure tibial load.

#### 4.2.1. Accelerometers and IMUs

Multiple studies [25,27,28,29,31,35,36] utilized accelerometers and/or accelerometers embedded in IMUs to measure tibia linear accelerations with respect to gravitational units (g) during walking and running. These sensors are a popular choice for biomechanical studies, due to their portability, flexible application, low power consumption and cost, and, as a result, their ease of use and versatility during data collection. They are also typically wireless and non-invasive, which makes them a popular technology for human-gait research.

Encarnación-Martínez, et al. [27] reported different tibial acceleration results from accelerometers than other studies [25,28,29,31,35,36], which may have been due to the sampling frequency selected for the accelerometers. The Nyquist theory states that the minimum sampling frequency should be twice the highest frequency present in a signal [53]. The research additionally states that human motion adds signal noise; therefore, a higher sampling frequency of 5 to 10 times the highest frequency is required. Thus, if 99% of the signal power captured when running is below 60 Hz, a required sampling frequency between 300 and 600 Hz should be employed [54]. Most studies [25,28,29,31,35,36] utilized sampling frequencies within their chosen accelerometers ranging from 1000 to 1600 Hz, which tends to be in line with most research [54]. Encarnación-Martínez et al. [27], utilized a threshold of 180 Hz, which was far lower than any other compared study, likely explaining the extremely low peak-acceleration values obtained from their results. As results between other studies using accelerometer data were still not perfectly correlated when compared to each other, as the sampling frequencies for all of these studies was at least 1000 Hz, it is unlikely that this can explain all of the result differences from study to study, and it remains more likely that the discrepancies can be explained by other variables (participants, surface, footwear, and foot strike pattern). The authors additionally noted that the cadence was significantly higher, and the stride lengths were significantly shorter when running on the treadmill, as opposed to overground, which likely contributed to the decreased joint moments and, therefore, changes in tibial acceleration.

Another issue presented with accelerometer measurements between studies which likely influenced the results was the measurement location (position). Of the studies that used accelerometers to measure tibial loading data [25,27,28,29,31,35,36], only two studies specified exactly where the accelerometers were positioned on the shank. Other studies [25,27,28,31] provided more vague locations, typically stated as the ‘distal and anteromedial aspect of the leg’. A systematic review on accelerometer placement in running gait analysis [55] highlighted key points for accelerometer placement, specifically, that they are placed close to the area of interest, and, ideally, on the distal tibia. Generally, when the sampling rates were 1000 to 1600 Hz, results were similar; only one study [27] was a large outlier regarding results, and this can likely be associated with the low (180 Hz) sampling rate that was used for their accelerometers.

#### 4.2.2. Strain Gauges

Milgrom et al. [32] assessed tensile and compressive forces on the tibia in the middle and distal thirds, comparing loads between walking and running utilizing instrumented bone staples with strain gauges as the data collection method. Strain gauges were a preferred method chosen to measure force in this study for reasons such as accurate strain localization, real-time measurements, and individualized strain data. Results highlighted greater peak axial-compression strain in the distal third of the tibia compared to the middle when walking and running, as well as an increase in 1.94 × peak axial-tensile strain when comparing walking to running.

#### 4.2.3. Three-Dimensional Motion Analysis

Three studies [30,33,34] utilized a camera system and a force-instrumented treadmill to obtain their data on tibial loading. Meardon et al. [30] examined the peak and per-step bone stress in recreational female and male runners during walking and running. Motion capture was used in this study, using retroreflective markers, allowing for the building of the musculoskeletal models which were used to estimate the internal forces acting on bones during walking and running. Kinetic data were obtained in the study using a force-instrumented treadmill that measured the ground reaction forces acting on the foot during ground contact. The combination of the kinetic and kinematic data collected was integrated to develop the musculoskeletal model that provided a simulation of how the muscles, bones and joints interacted during walking and running. Rice et al. [33] also utilized a motion camera system and retroreflective markers, as well as a force-instrumented treadmill for kinetic and kinematic data collection, which was then used to estimate how speed and steepness affect tibial loading during running. Rice et al. [34] again utilized a motion capture system and retroreflective markers, as well as a force-instrumented treadmill to measure both kinetic and kinematic data, which were then both integrated into a computerized model to provide estimations of peak and cumulative loading at different speeds and weight carriage.

Across the three studies, sampling rates for the force treadmills included 1000, 2000, and 1500 Hz [30,33,34], respectively. Modern treadmills often use sampling frequencies of 1000 Hz for sprint measurements, which has been referenced as an ideal frequency to ensure accurate capture of forces during sprinting [56]. Therefore, the selected sampling frequencies utilized in studies [30,33,34] were all likely capable of providing accurate force data. Additionally, the sampling frequencies of all motion capture cameras were 200, 200 and 300 Hz, respectively [30,33,34].

Although inverse dynamics has limitations in measuring joint forces, such as the absence of muscle activation, and its highly efficient computational nature can oversimplify underlying biomechanics [57], it promotes benefits such as efficiency and speed, it is enough for general joint-loading estimates, and it requires simpler data collection [57]. With these limitations in mind, all studies displayed an increase in tibial loads. For example, a 10–20% increase in running speed increased shear stress by 10 to 26% (note: WebPlot estimated) [30], and peak medial–lateral bending moment increased 11.2 Nm during faster speeds (3.1 m/s to 3.8 m/s) [34]. Caution should be taken when comparing these results to research done using alternate methods of data collection, due to the assumptions being made by the computerized model and generalized results. The lack of standardization was an issue when aiming to compare studies. What becomes an issue from this lack of standardization is that inconsistent results make it hard to provide load management recommendations to individuals involved in walking and running, regardless of whether it is for rehabilitation or training purposes.

### 4.3. Effects of Foot Strike Pattern, Speed and Gait Retraining

Clansey et al. [25] analysed the effects of tibial shock feedback and its ability to reduce impact loading using accelerometers. At the same speeds (3.7 m/s), the results showed that gait-retraining feedback resulted in a reduction in peak tibial acceleration by 31% after training, and a reduction of 22% was maintained one month after the training. Key factors facilitating this reduction noted by the authors included a change in their foot-strike pattern from rearfoot to midfoot, together with increased ankle plantarflexion and lower heel vertical velocity. This study highlighted the fact that, at higher speeds, when greater tibial loading is expected [10,25,26,27,28,29,30,31,32,33,34,35,36], gait-retraining feedback can allow for individuals to run at a higher speed with reductions in tibial load, alongside results that will likely remain after the retraining process [25]. Additional research [48] examining the effects of gait-retraining feedback on experienced runners, highlighted the fact that peak tibial acceleration decreased by 48% immediately and 44% at the one-month follow-up, at running speeds again at 3.7 m/s. Instead of measuring whether the foot strike pattern itself changed, results experienced after feedback, such as ‘running softer’ and ‘making footfalls quieter’, were given.

When analysing gait-retraining feedback and its effect on tibial loading, it appears that significant results can be expected [25,48]. Although as running speed increased, so did loading on the tibia, feedback was able to reduce overall tibial loading significantly post training, and the results also appeared to last a month after training. What is important about these results is that the running speeds were consistent, so for individuals potentially seeking to reduce tibial loading whilst also increasing or maintaining speed, gait-retraining feedback appears to be a viable option. Limitations to this idea are that individuals in the study who experienced gait-retraining feedback were chosen due to their pre-existing heel strike pattern; therefore, individuals who already have a mid-foot strike pattern likely will not have the same results.

### 4.4. Interactions Between Speed, Measurement Locations Surface, and Gait Retraining

The interactions between speed, measurement locations, surface and gait all have an influence on the impact of tibial loading, as evidenced by multiple studies [10,25,26,27,28,29,30,31,32,33,34,35,36]. Tibial loads increase as speed does, due to increased ground reaction forces and greater stress on the tibia. But these assessed loads vary, based on the measurement types used, such as accelerometers and their sampling frequency and placement on the body, as well as strain gauges versus motion-capture inverse dynamics. This section discusses the interactions between speed change, the measurement locations, the surface, and gait retraining on overall tibial loading.

Studies using accelerometers or IMUs [25,26,27,28,29,31,35,36] to measure tibial acceleration at different speeds consistently showed an increase in tibial loading as speed rises. Importantly, the exact magnitude of this loading depends on both accelerometer sampling frequency and accelerometer placement on the shank [25,26,27,28,29,31,35,36]. For example, Encarnación-Martínez [27] used a sampling frequency far below the other studies and, as a result, had tibial-loading values significantly lower than the respective studies it was compared to. Additionally, Milgrom et al. [32] utilized strain gauges to examine strain at the middle and distal thirds of the tibia, and reported significantly greater tibial loads distally. However, the tibial measurement locations (positions) were not standardized, so comparisons between studies are difficult, as lower accelerometer placement will likely pick up higher frequencies of load.

The type of surface chosen also played a critical role in determining tibial loading. A study [27] reported consistently higher tibial loading when running on a non-motorized, compared to a motorized, treadmill. Due to Johnson et al. [28] reporting greater tibial loading during overground running than on motorized treadmills, it would likely be believed that the non-motorized treadmill may elicit similar loading results, due to individuals having to self-produce force, but results reported greater tibial loading for the motorized treadmill. The authors hypothesized that the difference in tibial loading could have been caused by the curved, concave belt, which caused a forward-lean position favouring forefoot instead of midfoot and heel striking patterns [27]. This illustrates additional considerations regarding tibial loading, as different surfaces, and postures can clearly affect the magnitude of force experienced by the tibia.

Gait retraining was shown to significantly reduce the loads exerted on the tibia, even at the same running speeds. Two studies [25,48] examined tibial loading at different speeds of movement, and the effects that gait retraining would exhibit. Both studies reported significantly reduced tibial loading at the same measured speeds (↓22% and ↓48%), and this was reported by one study as being likely due to the runners adopting a more midfoot strike pattern instead of the heel strike pattern.

Interaction between the variables showed that speed was the primary tested contributor to increased tibial loading during human movement. Importantly, it is challenging to make direct comparisons between studies, as different measurements taken can significantly affect the results. As increased speed amassed tibial loading, the surface, or running posture being used could also either increase or decrease the load on the tibia, although it would remain safe to assume that if speed were increasing per individual on the same surface with an unchanged running pattern, tibial loads would likely follow with an increase. Additionally, running gait also appeared to influence the tibial-loading results, as gait retraining showed promising results at being able to reduce tibial loads at the same respective running speeds by altering running mechanics.

### 4.5. Limitations

This scoping review sought to identify prevalent methods to measure (and current knowledge of) tibia loads during human gait on different surfaces. A more systematic review method for this population, concept and context (PCC), with critical appraisal, is optional for scoping reviews [18] but was also not possible here, due to the limited number of scientific studies identified and due to the heterogeneity of the methods employed. While limiting the search to EMBASE and MEDLINE may have missed eligible studies, reference lists of included studies were screened to identify additional eligible publications. Data reported in figures by Milgrom et al. [32] was extracted by a single author using WebPlotDigitiser software. Whilst repeat digitization, including the calibration step, indicates very high intra-rater reliability, the accuracy of the extraction is untested. For that reason, patterns and trends are described without cross-study quantitative synthesis. The small volume of studies available on this topic is likely due to the invasive direct (in vivo strain gauge) and/or time-consuming indirect model-derived (FP+3DMA and/or 3DMA+MSS modelling) methods available. Wearable sensor technology such as small, lightweight IMUs may accelerate scientific study on this topic, but the results to date are difficult to summarize, due to the non-standardization of methods (e.g., sensor position [58]). Wearable sensor methods may also under- or over-estimate the biomechanical load due to soft-tissue artifact, even in more bony regions such as the distal tibia [59]. Finally, it should be acknowledged that any indirect methods of measuring impact loads through the tibia may not adequately indicate the internal tibial bone stress, due to the contribution of the soft tissues (e.g., muscle forces) and method or modelling assumptions.

## 5. Conclusions

Increased human-gait speed was consistently associated with higher impact-related metrics of tibial loads. This was consistent across all studies analysed in this scoping review, despite the varied direct (in vivo strain gauges) and indirect, external surrogate (accelerometers, IMUs) and model-derived (3DMA, FP+3DMA [inverse dynamics]) measurement methods. Despite this clear pattern, it is important to note that results from the different methods identified are not inherently equivalent. Direct comparisons of results between methods are, therefore, inappropriate. Discrepancies in results between studies that used the same impact metrics highlight the importance of standardized methods, as multiple factors can affect the results of direct and indirect tibial-load measures.

In addition to varying hardware for data collection, methods used even for the same hardware also varied. For example, accelerometer/IMU placement varied and was frequently omitted from reports. Additionally, data analysis methods (e.g., data filtering, tibial-acceleration-signal measure) were inconsistent. Different methods of using hardware also make it challenging to compare results accurately and coherently.

Additionally, surface was also shown to modulate the tibial-load patterns, but was often not reported. Differences in tibial-acceleration loads across overground, motorized-treadmill, and non-motorized-treadmill running conditions have been reported in certain studies, but these findings are dependent on the experimental protocol, running velocity assessed, and measurement methods. Within that limitation, these differences were likely influenced by biomechanical adaptations to accommodate the surfaces which may include changes to running technique such as a more mid-to-forefoot striking posture when running on the non-motorized treadmill [60,61].

Gait mode, speed, and surface all likely influence impact loads. For that reason, recommended reporting standards are provided (Table S2). Clinicians and coaches should use this information cautiously when seeking to modify exercises or to develop a rehabilitation plan, with a primary focus on tibia loads. The tibial-loading datasets are obscured by a small evidence base (12 studies) and non-standardized (heterogeneous) measurements, and should be used with caution when applied to real-world scenarios. Future research should establish standardized measurements for similar types of studies (e.g., accelerometers/IMUs, inverse dynamics), especially when examining changes to tibial-load patterns prospectively, during gait interventions (e.g., gait retraining).

## Figures and Tables

**Table 1:** Summary of studies investigating biomechanical measures of tibia load in human gait: purpose, study outcomes, strengths, and limitations.

Reference	Purpose	Study Outcomes	Strengths	Limitations
Clansey, Hanlon, Wallace, Nevill and Lake [25]	To identify the effects of real-time gait retraining feedback on impact loads and economy during overground running.	No changes to running economy or vertical impact peak GRF. Reduced tibial axial acceleration following intervention, which was maintained 1 month post. Reduced GRF loading rate following intervention but not maintained 1 month post.	Well-defined participant inclusion criteria. Intervention study with a control group. Inter-disciplinary methods: biomechanics and physiology. Triaxial accelerometer with high sampling rate (1500 Hz). Justifiably defined extracted measures from accelerometer. Combination of traditional statistical significance and magnitude-based differences utilised. Real-time visual and auditory feedback system for gait retraining.	Small sample size in each group (*n* = 10–12). Exclusion of females. Indoor, laboratory environment. Footwear and testing surface not reported Absolute sensor position; not adjusted for participant tibia length. Sensor fixation not reported. Accelerometer measurement threshold not reported. Short intervention period of 3 weeks. Short post-training follow-up retest of 1 month. No reliability measures reported. No normality check of the dataset prior to the statistical tests.
D’Angeli, Belvedere, Ortolani, Giannini and Leardini [26]	To provide a comprehensive dataset on load (GRF and moments) along 10 points of the tibial shaft in healthy, young adults, for a range of physical activities.	Load patterns were different between the anatomical axes, walking speeds, and along the tibial shaft. Variability in peak tibial moments was high, especially when asked to walk slower than their preferred pace, and at the tibial plateau. Positive relationship between walking speed and tibial forces and moments.	Measures of both male and females. Detailed dataset on load along the tibial shaft during movement. Normalisation of measures with respect to body weight and height.	Indoor, laboratory environment. Footwear and testing surface not reported. Walking distance not reported. Walking speeds were not quantified for the three tasks measured: low, self-selected, high. Sensor fixation not reported. Low sampling rate for inverse dynamics measures (100 Hz). Filtering method not reported. No normality check of the dataset prior to the statistical tests. Limited statistical analyses of the dataset.
Encarnación-Martínez, Catalá-Vilaplana, Berenguer-Vidal, Sanchis-Sanchis, Ochoa-Puig and Pérez-Soriano [27]	To analyse impact accelerations and spatiotemporal parameters during running on curved non-motorised and motorised treadmills.	No significant differences between men and women for the gait metrics examined. Reduced tibial acceleration magnitude (difference between positive and negative peaks), tibial acceleration magnitude, and head acceleration rate when running on the curved, non-motorised treadmill.	Measures of both males and females. Inter-disciplinary methods: biomechanics and physiology. Triaxial accelerometer. Confidence intervals and Cohen’s effect sizes reported for significant differences detected.	Low proportion of females (19%) in dataset. Sensor position poorly described. Higher sensor mass (50 gm). Low sampling frequency for accelerometers (180 Hz). Footwear not controlled. No reliability measures reported. Bonferroni adjustments may have introduced Type II error [37].
Johnson, Outerleys, Jamison, Tenforde, Ruder and Davis [28]	To compare IMU measures of tibial linear accelerations between motorised treadmill and various stages of overground, road marathon running.	Mean vertical and resultant PTA was lower when running on a treadmill compared to all stages of overground marathon running. Measures of PTA during treadmill running are only able to predict approximately 41% of the variance of overground marathon running. Vertical and resultant PTA was highest during the early stage of marathon running when the runners were less fatigued and running faster (12th km: 3.35 m/s, 15.72 g → 40th km: 2.92 m/s, 13.61 g).	Large sample size. Measures of both males and females. Low mass (12 gm), triaxial accelerometer. Good sampling frequency (1000 Hz). Ecologically valid dataset of IMU measures during road marathon running. Repeated measures during different stages of a full marathon race.	Using the term “tibial shock” to describe peak tibial accelerations may not be appropriate. See methods for measuring shock (e.g., Refs. [31,38]). Absolute sensor position: not adjusted for participant tibia length. Low IMU sampling threshold (16 g) for overground running with author reports of data clipping and interpolation methods employed. Footwear not controlled across participants. Reliability of within-lab measures not reported. Bonferroni adjustments may have introduced Type II error [37]. While statistically significant, the variance explained by speed alone was low (R^2^ = 0.09–0.27), so could not accurately model vertical or resultant tibial shock (NB: these results were therefore not reported in Table 3 of this paper). No examination of the effect of foot strike pattern on the PTA results.
Johnson, Outerleys and Davis [29]	To establish whether tibial accelerations in the medial–lateral and anterior directions are related to loading rates in the same directions during motorised treadmill running at a self-selected speed.	High variability for all measures. Large IQR and range, often higher than median, for both accelerometer and force plate results. Peak tibial accelerations (PTAs) in the medial axis were strongly related to peak lateral loading rate [LR; R_s_(16) = 0.86, *p* < 0.001]. PTAs in the lateral axis were strongly related to peak medial LR [R_s_(16) = 0.91, *p* < 0.001]. PTAs in the anterior axis were moderately related to peak posterior LR [R_s_(16) = 0.51, *p* < 0.030]. When the tibia accelerates medially, the GRF acts in the opposite direction (laterally), and vice versa. Low magnitude of GRFs and PTAs on the medial–lateral and anterior–posterior axes, but may provide insight on adaptation to changes in internal (e.g., fatigue) and external (e.g., surfaces during trail running) factors.	Explored less-researched GRF and PTA axes. Low mass (9.5 gm), triaxial accelerometer. High sensor range (200 g) and sampling rate (1600 Hz). Non-parametric statistics partially employed, due to the detection of non-normally distributed data.	Exclusion of males. GRF and IMU data collection not synchronised. Absolute sensor position: not adjusted for participant tibia length. Footwear not reported. Posterior acceleration peak and second posterior instantaneous braking peak not assessed. Reliability of within-lab measures not reported. Bonferroni adjustments may have introduced Type II error [37]. Strength of relationships from Spearman rank coefficients assessed using parametric correlations and Cohen’s effect size statistics for non-normally distributed dataset.
Meardon, Derrick, Willson, Baggaley, Steinbaker, Marshall et al. [30]	To examine tibial stress during motorised treadmill during various gait tasks (walk, preferred run, slow run, fast run) in male and female recreational runners.	Peak tibial compression, tension and shear stress increased with gait speed for both men and women, especially when transitioning between walking and running (2-fold increase). Women sustained greater compression, tension and shear stress than men, due to their smaller bone geometry; greater tibia forces in the medial–lateral axis. Men had larger bone geometry and bone strength indices, higher tibia forces in the axial axis and anterior–posterior bending moments. Women may need slower progression when commencing and progressing with running programs, to reduce the risk of bone stress injury.	Good sample size. Measures of both males and females. Inclusion of Bone-specific Physical Activity Questionnaire (BPAQ) for participant descriptors. Footwear controlled (standardised). Detailed marker set. Inclusion of axial MRI scans in modelling.	Low sampling frequency for kinematics component (200 Hz) of inverse dynamics for tibial stress measures during running. No reliability measures reported. Musculoskeletal modelling assumptions. Bonferroni adjustments may have introduced Type II error [37].
Mercer, Vance, Hreljac and Hamill [31]	To investigate the characteristics of shock attenuation between the distal tibia and forehead during sub-(50, 60, 70, 80, 90%) and maximal (100%) speed running on a motorized treadmill.	Tibial accelerations and shock attenuation increased (note: indicated by lower ratios) significantly with treadmill speed for male runners. Strong correlations between stride length and running speed (r = 0.92), and stride length and shock attenuation (r = 0.71). Changes in shock attenuation with running speed were due to changes in stride length and leg accelerations; that is, the head accelerations were predominantly stable for the runners tested, to maintain a stable visual field [39]. The authors noted that one participant (out of eight) responded differently to the group. Their shock attenuation did not change with speed. No case study analysis was performed to further describe that result.	Low mass accelerometer (2 gm). Shock attenuation measures from power spectral density analyses. Group and individual analyses.	Small sample size (*n* = 8). Exclusion of females. Treadmill used not reported. Footwear not reported. Uniaxial accelerometer. No external load measures. No reliability measures reported.
Milgrom, Voloshin, Novack, Milgrom, Ekenman and Finestone [32]	To identify the inter-participant variance of in vivo medial tibial strains during exertional physical activities. To better understand how in vivo medial strains might reflect susceptibility to medial tibial stress fracture.	In vivo strain magnitudes were higher than previously reported from finite element modelling [40]. Running produced 1.94 greater tensile strains when compared to walking (*p* = 0.009). During treadmill running the distal tibia experienced significantly greater compression strains than the mid tibia, for all cases (*p* < 0.001). Individual (case study) patterns emerged when comparing distal- and mid-tibia strains for peak axial compression when walking, and peak axial tension when walking and running. This may explain the variation in the anatomical location of tibial stress fractures in athletes (and defence personnel) when completing the same training program.	Footwear controlled (standardised). Measures directly on bone. Combination of traditional statistical significance and magnitude-based differences utilised. Group and individual analyses.	Very small sample size (*n* = 3), but likely due to invasive protocol. Exclusion of females. Treadmill used not reported. Model and type of running shoes not reported. Reliability of in vivo measures not reported.
Rice, Kurz, Mai, Robertz, Bill, Derrick and Willwacher [33]	To quantify tibial bending moments and stress at the anterior and posterior peripheries when running on a motorised treadmill at different speeds (2.5, 3.0, 3.5 m/s) and gradients (−15, −10, −5, 0, +5, +10, +15%).	Changes to treadmill running speed and gradient have an independent effect on the internal tibial loads at the distal third. Greater running speed resulted in higher peak tibial bending moments and peak tibial stress in both the anterior and posterior regions. In the medial–lateral direction, the distal tibia loads were mostly negative during stance, generating tensile stress in the anterior tibia and compression stress in the posterior tibia (concave loading pattern). To decrease internal distal tibia loads, prescription should include slow treadmill running downhill at a gradient of 10^0^ or greater. Vice versa when prescribing treadmill running exercise to increase internal distal tibia loads. These guidelines may provide runners with strategies to minimise the risk of tibial stress injury.	Measures of both males and females. Detailed marker set. Statistical parametric mapping (SPM) analyses utilised to examine the loading pattern during stance.	Female data likely underpowered, due to small sample size (*n* = 9). Footwear not controlled across participants. Low sampling frequency for kinematics component (200 Hz) of inverse dynamics for tibial mechanics measures during running. Musculoskeletal modelling assumptions, but direct validation of the approaches is not possible. No reliability measures reported. Bonferroni adjustments may have introduced Type II error [37].
Rice, Seynnes and Werkhausen [34]	To investigate the effects of speed (preferred, preferred + 20%) and weight carriage (0, +20% body weight) on peak and cumulative tibia loads in motorised treadmill running.	Running at a 20% faster speed increased the peak and cumulative loads per kilometre by 8.0 and 4.8%, respectively. Carriage of an additional 20% of body weight via a weighted vest increased cumulative loads per kilometre by 6.6 and 8.5%, respectively. There were no interaction effects of running speed and weight carriage on the tibia loads. The timing of the peak bending moment of the distal tibia was delayed by weight carriage (approx. unweighted 62% → weighted 66% of stance).		Exclusion of females. Barefoot running may not be the same as when shod. Modelling assumptions. Musculoskeletal modelling assumptions, but direct validation of the approaches is not possible. No reliability measures reported. Bonferroni adjustments may have introduced Type II error [37].
Sheerin, Besier and Reid [35]	To investigate the impact of treadmill running velocity (2.7, 3.0, 3.3, 3.7 m/s) on peak resultant tibial acceleration in habitual, injury-free runners.	An average increase of 3.8 g (38%) in PTA was identified between the slowest (2.7 m/s) and fastest (3.7 m/s) treadmill running tests. Moderate linear relationship between running velocity and resultant PTA, explaining 19% of the variation in the PTAs observed. Resultant PTAs during running are likely affected by many individual factors, and therefore a personalised approach to understanding how each runner responds to running exercise is needed. Low inter-limb asymmetry was observed in the resultant PTA for all speeds in the group of runners (<6%).	Large sample size. Measures of both males and females. Footwear controlled (standardised). Low mass (12 gm), triaxial accelerometers. Combination of traditional statistical significance and magnitude-based differences utilised.	Bonferroni adjustments may have introduced Type II error [37].
Voloshin [36]	To analyse the effect of speed (0.89, 1.12, 1.34, 1.57, 1.79 m/s) on the linear axial acceleration of the tibia tuberosity at heel strike during treadmill walking of young, healthy adults.	Near-linear relation between treadmill walking speed and tibial acceleration (proximal). Accelerations increased five times faster than GRFs with increasing treadmill walking speed.	Low mass accelerometer (2.3 gm).	Exclusion of females. Uniaxial accelerometer. Potential movement artefacts due to method of sensor fixation. No reliability results reported.

**Table 2:** Summary of studies investigating biomechanical measures of tibia load in human gait: participant details, inclusion criteria, gait mode and speed assessed, environment, surface and footwear used, tibia load measurement methods, and sensor and/or retroreflective marker protocol (position, attachment method).

Reference	Participant Details/Sample Size	Inclusion Criteria	Gait Mode and Speed Assessed	Environment, Surface and Footwear Used	Tibia Load Measurement Methods	Sensor and/or Retroreflective Marker Protocol
Clansey, Hanlon, Wallace, Nevill and Lake [25]	Healthy adult recreational rearfoot striking runners (male: *n* = 22; control group: *n* = 12, age = 34 ± 11 years, height = 1.8 ± 0.1 m, body mass = 75.1 ± 6.9 kg; intervention group: *n* = 10; age = 33 ± 9 years, height = 1.8 ± 0.1 m, body mass = 77.2 ± 11 kg).	Running at least 30 km/week and injury-free for 6 months. No history of major MSS injury or cardiovascular pathology. Rearfoot striking pattern of running. Peak tibial axial (PTA) loads >9 g during pre-screening assessment.	Running overground for 15 m. 3.7 m/s.	Indoor, laboratory. Surface not reported. Footwear not reported.	Triaxial accelerometer (embedded in wireless EMG sensor, TeleMyo 2400T G2, Noraxon, Scottsdale, AZ, USA; mass = 2.8 gm, sensitivity rage = ±0.67 V/g). Sampling at 1500 Hz. Fourth order, low pass Butterworth filter 60 Hz. Reliability not reported. No normality check of the dataset.	Unilateral; right limb only. Distal anteromedial aspect of tibial bone. Approximately 0.10 m above the ankle joint centre [41]. Axial axis aligned with long axis of the tibial bone. Skin-mounted. Skin stretched with water -epellent adhesive tape (kinesiology tape, Vivomed, UK) before sensor placement. Sensor fixation not reported.
D’Angeli, Belvedere, Ortolani, Giannini and Leardini [26]	Healthy adults (overall: *n* = 20, age = 27 ± 4 years, height = 1.7 ± 0.1 m, body mass = 63.6 ± 14.8 kg; males: *n* = 9; females: *n* = 11).	Not stated.	Walking overground; distance not stated. Low, self-selected, and high speeds; exact velocity not reported.	Indoor laboratory. Surface not reported. Footwear not reported.	Eight-camera 3DMA (Vicon Motion Systems, Oxford, UK). Two force platforms (Model not stated, Kistler, AG, Winterthur, Switzerland). Sampling at 100 Hz. Inverse Dynamics: 3D moments calculated via vector product of the 3D position vector of tibial shaft marker and the 3D GRF vector. Filtering not reported. Reliability not reported. No normality check of the dataset.	Unilateral; dominant limb only (Right = 19/20). Lower limb marker set of 20 retroreflective markers [42], plus 10 additional markers along the tibial shaft. Skin-mounted. Tibia/fibula frontal plane defined as the line passing between the midline between the ankle malleoli and the head of the fibula. Marker fixation not reported.
Encarnación-Martínez, Catalá-Vilaplana, Berenguer-Vidal, Sanchis-Sanchis, Ochoa-Puig and Pérez-Soriano [27]	Healthy adult recreational runners (overall: *n* = 27, age = 25 ± 7 years, height = 1.7 ± 0.1 m, body mass = 64.4 ± 10.3 kg; males: *n* = 22; females: *n* = 5).	Physically active; run a minimum of twice per week. Training volume of 20 km per week or more. Not overweight or obese (BMI > 24.9 kg/m^2^). No lower limb injuries in last six months. No history of heart failure. No neurological or musculoskeletal disorders affecting normal gait. Not taking medication that interferes with stability during running.	Treadmill running. +1% incline both treadmills. 8 min of each: 2.77 m/s, 3.33 m/s, and self-selected speed in randomized order. Self-selected speed: Treadmill 1 = 2.73 ± 0.02 m/s, Treadmill 2 = 2.82 ± 0.02 m/s [Estimated from reported stride length and stride frequency results].	Indoor laboratory. Curved non-motorised treadmill (Treadmill 1: ZRO = T, Bodytone International Sport S.L., Molina del Segura, Spain; belt—length = 150 cm, width = 40 cm). Motorised treadmill (Treadmill 2: pulsar ^®^ 3p, h/p/cosmos sports & medical gmbh., Nuβdorf, Germany; belt—length = 190 cm, width = 65 cm). Participants’ own footwear.	Triaxial accelerometer (Pikkulab, Blantic Design, Valencia, Spain; mass = 50 gm, 50 × 20 × 10 mm, range = ±16 g). Sampling at 180 Hz. Second order, low-pass Butterworth filter 50 Hz Reliability not reported. Normality check using Shapiro–Wilk test.	Unilateral; limb not stated. Distal and anteromedial portion (proximal) tibia. Forehead. Skin-mounted. Double-side adhesive tape. Secured with elastic straps/belts.
Johnson, Outerleys, Jamison, Tenforde, Ruder and Davis [28]	Marathon runners (overall: *n* = 192, age = 45 ± 11 years, height = NR, body mass = NR kg; males: *n* = 105; females: *n* = 87). Mix of recreational and more competitive runners.	Registered and participated in the 2016 marathon race. 18 years or older No current musculoskeletal injury. No conditions known to affect gait, balance or sensorimotor function. Compliance and/or successful completion of study data collection protocols (complete data from IMU for treadmill & outdoor, wore the IMU on race day correctly, wearing IMU correctly did not cause discomfort/pain, wore same shoes for all tests).	Treadmill running at 90% of self-reported, projected race speed (3.06 ± 0.38 m/s). 30 s of running; approximately 35 strides each leg. Overground running for entire marathon race (12th km: 3.35 ± 0.45 m/s; 23rd km: 3.21 ± 0.46 m/s; 40th km: 2.92 ± 0.51 m/s).	Indoor laboratory Motorised treadmill (4Front, Woodway, Waukesha, WI, USA; belt—length = 183 cm, width = 89 cm). Footwear not reported, but required to wear same footwear for both tests. Outdoor environment. Race location, surfaces, terrain, and weather conditions not reported.	Triaxial accelerometer (Blue Thunder, iMeasureU, Auckland, New Zealand; 40 × 28 × 15 mm, mass = 12 gm, range = 16 g). Sampling at 1000 Hz. Second-order, band pass Butterworth filter (0.4 Hz, 10 Hz). Reliability cited from external laboratory/research group [43]. Normality check using Shapiro–Wilk test.	Unilateral, right limb only. Distal–medial tibia IMU *y*-axis aligned with the long axis of the tibia, where positive y accelerations were directed towards the proximal tibia and positive xaccelerations directed posteriorly. Skin-mounted. Adjustable strap. Strap tightened as much as possible without causing discomfort.
Johnson, Outerleys and Davis [29]	Healthy adult recreational rearfoot striking runners (female: *n* = 18, age = 33 ± 11 years, height = 1.7 ± 0.1 m, body mass = 72.4 ± 15.4 kg).	Habitual runners in previous 6 months of at least 5 miles per week (average = 20 ± 13 miles/week). No injuries in previous 3 months. Free of prior surgery or conditions that would affect gait. Comfortable running on a treadmill. Rearfoot striking pattern of running (contacting the ground with the heel first; confirmed via high-speed video).	Treadmill running at a self-selected speed (2.97 ± 0.31 m/s). 16 s of running; approximately 20 strides each leg.	Indoor laboratory. Motorised, instrumented treadmill (AMTI, Watertown, MA, USA, 1500 Hz; belt—length = 152 cm, width = 64 cm). Footwear not reported.	Triaxial accelerometer (Blue Trident, iMeasureU, Auckland, New Zealand; 42 × 27 × 11 mm, mass = 9.5 gm, range = 200 g). Sampling at 1600 Hz. Second-order, band pass Butterworth filter. (0.4 Hz, 10 Hz) Reliability cited from external laboratory/research group [44,45]. Normality check using Shapiro–Wilk test.	Unilateral; right limb only. Distal–medial tibia, 1 cm above the superior border of the malleolus. Skin-mounted. Double-sided tape. Kinesio tape. Elastic wrap (SuperWrap^TM^, Fabrifoam, Exton, PA, USA).
Meardon, Derrick, Willson, Baggaley, Steinbaker, Marshall and al [30]	Healthy adult runners (overall: *n* = 40, height = 1.8 ± 0.1 m, body mass = 80.1 ± 12.7 kg; males: *n* = 20, age = 25 [23,24,25,26,27] years; females: *n* = 20, age = 55 [23,24,25,26] years).	Aged 18 to 35 years Running more than 16 km per week or more at the time of the study. Treadmill comfort score greater than 7 out of 10; indicative of completely comfortable. No current musculoskeletal injury or pain with activity. No neuromuscular or cardiopulmonary conditions that could impair normal running function. No contraindications to magnetic resonance imaging.	Treadmill gait at 4 speed: (1) walking (1.3 m/s), (2) running (preferred speed), (3) slow running (90% of preferred), (4) fast running (110% of preferred). Preferred running speed: males = 2.97 [2.89–3.05] m/s, females = 2.79 [2.66–2.92] m/s. 15 s of gait for each speed.	Indoor laboratory Motorised instrumented dual-belt treadmill (Bertec, Columbus, OH, USA; belt—length = 175 cm, width = 100 cm). Standardised shoes for testing (ProGrid Ride, Saucony).	Ten-camera 3DMA (Qualisys, Kvarnbergsgatan, Göteborg, Sweden). Motorised instrumented dual-belt treadmill (Bertec, Columbus, OH, USA). Sampling at 200 Hz for kinematics and 1000 Hz for kinetics. MRI of the right tibia (1.5 T scanner with a torso coil, Achieva, Phillips, Amsterdam, Netherlands). Inverse Dynamics and static optimisation: 3D moments, joint reaction forces, and internal muscle forces. Bone geometry and bending and torsional bone-strength indices determined from axial MRI scans. Finite-element mesh methods for bone stress modelling. Fourth-order zero-lag low-pass Butterworth filter 15 Hz. Reliability not reported. Method of assessing normality of the dataset not reported.	Unilateral; right limb only. Lower limb and trunk marker set of 55 retroreflective markers [46]. Marker-fixation method not reported.
Mercer, Vance, Hreljac and Hamill [31]	Physically active adults (male, *n* = 8, age = 25 ± 5 years height = 1.8 ± 0.6 m, body mass = 80.0 ± 8.9 kg).	Physically active Free from lower-extremity injury at the time of testing. Experienced in running on a treadmill.	Treadmill running at 50, 60, 70, 80, 90 and 100% (3.2 ± 0.3, 3.8 ± 0.3, 4.5 ± 0.4, 5.1 ± 0.4, 5.7 ± 0.5, and 6.4 ± 0.5 m/s) of maximum speed able to be sustained for the test. 20 s of running.	Indoor laboratory. Motorised treadmill (details not reported). Footwear not reported.	Uniaxial accelerometers (353C67, PCB Piezoelectronics, Depew, NY, USA; mass = 2 gm, sensitivity = 100 mV/g). Sampling at 1000 Hz. Fourth-order, low-pass, zero-lag Butterworth filter 100 Hz. Reliability not reported. No normality check of the dataset.	Unilateral; limb not stated. Distal antero–medial aspect of the leg. Anterior aspect of the forehead. Leg accelerometer mounted onto a small piece of balsa wood. Leg accelerometer secured using an elastic Velcro strap. Head accelerometer secured to rigid plastic headgear using wax supplied by the manufacturer.
Milgrom, Voloshin, Novack, Milgrom, Ekenman and Finestone [32]	Physically active adults (male, *n* = 3, age = 36 [29,30,31,32,33,34,35,36,37,38,39,40] years height = 1.8 ± 0.1 m, body mass = 80.7 ± 1.5 kg).	Male. Aged between 21 and 40 years. Physically active lifestyle. No history of orthopaedic knee or leg problems.	Treadmill walking (1.39 m/s) and running (3.61 m/s).	Indoor laboratory. Motorised treadmill (details not reported). Nike running shoes (model not reported).	Bone staples (CONMED, Utica, New York, NY, USA; 16 × 15 mm) instrumented strain gauge (EA-06-031DE-350, Measurements Group, Raleigh, NC, USA) bonded to the undersurface of the staple. Sampling at 1000 Hz. Fast Fourier transformation 30 Hz. Reliability cited from same laboratory/research group, but for surface strain gauges [47]. Normality check using Shapiro–Wilk test.	Unilateral; left limb only. Strain gauges surgically implanted under local anaesthesia. Aligned along the long axis of the tibia, on the flat medial aspect, closer to the posterior than anterior border, (1) centred over the mid diaphysis (proximal strain gauge) and (2) centred at the distal third of the tibia (distal strain gauge).
Rice, Kurz, Mai, Robertz, Bill, Derrick and Willwacher [33]	Healthy adult recreational runners (overall: *n* = 20, age = 24 ± 4 years, height = 1.8 ± 0.1 m, body mass = 67.8.1 ± 4.0 kg; males: *n* = 11; females: *n* = 9).	Older than 18 years. Run at least 10 km per week. No running-related overuse injury within the last year.	Treadmill running at 2.5, 3.0 and 3.5 m/s. Level running at the three speeds. Running at the three speeds for gradients of +5, +10, and +15% (uphill) and −5, −10, and −15% (downhill).	Indoor laboratory. Motorised instrumented treadmill (Gaitway 3D, HP Cosmos, Traunstein, Germany; belt—length = 220 cm, width = 95 cm). Participants’ own footwear.	17-camera 3DMA (Type 5+ and 700, Qualisys, Goteborg, Sweden). Motorised instrumented treadmill (Gaitway 3D, HP Cosmos, Traunstein, Germany). Sampling at 200 Hz for kinematics and 2000 Hz for kinetics. Inverse Dynamics: muscular forces from 11 muscles, joint reaction forces, resultant bending moments and stress. Fourth-order, recursive, low-pass Butterworth filter 20 Hz. Reliability not reported. No normality check of the dataset.	Bilateral; unclear if both limbs were reported in results. Full-marker body set of 82 retroreflective markers, including various anatomical landmarks and four-marker tracking clusters.
Rice, Seynnes and Werkhausen [34]	Adult male recreational distance runners (*n* = 14; age = 27.3 ± 4.4 years, height = 1.8 ± 0.1 m, body mass = 68.1 ± 6.3 kg).	Injury-free at the time of testing. Running at least 40 km/week.	Treadmill running at preferred speed (3.1 ± 0.3 m/s) and preferred speed +20% (3.8 ± 0.4 m/s). Preferred speed defined as the speed which participants could comfortably sustain for 1 h of running. Body weight only and with 1–2 weight vests (10 kg each), equivalent to +20% of their body weight.	Indoor laboratory. Motorised instrumented treadmill (M-Gait, Motekforce Link, Amsterdam, the Netherlands, belt—length = 180 cm, width = 108 cm). Barefoot.	11 camera 3DMA (Qualisys, Gotenburg, Sweden) sampling at 300 Hz. GRF data captured from an instrumented treadmill (M-Gait) sampling at 1500 Hz. Seven steps of data for each condition for each participant. Second -rder Butterworth filter 15 Hz. Inverse dynamics: muscular forces for 11 muscles of the lower extremity, tibial bending moments, cumulative-weighted impulse. Reliability not reported. No normality check of the dataset.	Unilateral; left limb only. Hip/pelvis and right leg marker set of 20 reflective markers, including various anatomical landmarks and 4-marker tracking clusters.
Sheerin, Besier and Reid [35]	Adult recreational distance runners (overall: *n* = 85, age = 40 ± 9 years, height = 1.8 ± 0.1 m, body mass = 73.9 ± 11.0 kg; males: *n* = 65; females: *n* = 20).	Injury-free at the time of testing. Running at least 20 km/week. Running at least three or more times per week. Running for 6 months or more.	Treadmill running in 2-min increments at 2.7, 3.0, 3.3 and 3.7 m/s.	Indoor laboratory. Motorised instrumented treadmill (Bertec, Columbus, OH, USA, belt—length = 175 cm, width = 100 cm). Standardised neutral running shoes (Kudrow, Asics, Kobe, Japan).	Triaxial accelerometers (Blue Thunder, iMeasureU, Auckland, New Zealand, 40 × 28 × 15 mm, mass = 12 gm, range = 16 g). Sampling at 1000 Hz. Fourth-order, dual low-pass Butterworth filter 60 Hz. Reliability reported in introduction from in-house research [44]. No normality check of the dataset.	Bilateral. Distal anteromedial tibia. 3 cm superior to the crest of the medial malleolus. First attached with double-sided tape. Secondly, wrapped tightly with elastic adhesive bandage (Amtech, Auckland, New Zealand).
Voloshin [36]	Healthy male adults (*n* = 15, age = 20 ± 1 years, height = 1.8 ± 0.1 m, body mass = 73.3 ± 8.6 kg).	Healthy. Participating in calisthenics twice per week. Familiar and comfortable with treadmill exercise. No previous history of muscle weakness, neurological disease, or drug therapy.	Treadmill walking at 0.89, 1.12, 1.34, 1.57 and 1.79 m/s.	Motorised treadmill (Star Trac, Irvine, CA, USA, belt—length = 152 cm, width = 55 cm).	Uniaxial accelerometer (A303, PCB, Depew, NY, USA, mass = 2.3 gm). Sampling at 1000 Hz. 16 s data capture; approximately 16 heel strikes. No filter reported. Some participants performed the test twice to assess the repeatability (reliability) of the measures, but results not reported. No normality check of the dataset.	Unilateral; right limb only. Tibial tuberosity. Axial axis of the sensor aligned to the longitudinal axis of the tibia. Attached externally to a metal holder. Metal holder trapped to the limb as tightly as possible, without causing discomfort, using Velcro strips.

**Table 3:** Summary of studies investigating biomechanical measures of tibia load in human gait: tibial and other load results (kinetics and kinematics).

Reference	Tibial Load Results	Other Impact Load Results: Kinetics	Other Impact Load Results: Kinematics
Clansey, Hanlon, Wallace, Nevill and Lake [25]	Data reported as average ± SD ***Repeated measures of control group*** Peak Tibial Axial Acceleration = 9.78 ± 1.68 g (baseline), 9.99 ± 1.97 (3 weeks later), 9.68 ± 1.87 g (7 weeks later). ***Repeated measures of intervention group*** Peak Tibial Axial Acceleration = 10.67 ± 1.85 g (baseline), 7.39 ± 1.48 (3 weeks later), 8.30 ± 1.82 g (7 weeks later).	Data reported as average ± SD. ***Repeated measures of control group*** Vertical Impact Peak GRF = 2.51 ± 0.16 BW (baseline), 2.53 ± 0.16 BW (3 weeks later), 2.54 ± 0.15 BW (7 weeks later). Vertical instantaneous GRF loading rate = 124.61 ± 26.97 BW/s (baseline), 137.37 ± 46.93 BW/s (3 weeks later), 123.18 ± 35.56 BW/s (7 weeks later). ***Repeated measures of intervention group*** Vertical Impact Peak GRF = 2.72 ± 0.17 BW (baseline), 2.62 ± 0.16 BW (3 weeks later), 2.59 ± 0.19 BW (7 weeks later). Vertical instantaneous GRF loading rate = 113.87 ± 33.01 BW/s (baseline), 92.10 ± 27.06 BW/s (3 weeks later), 101.63 ± 36.76 BW/s (7 weeks later).	Data reported as average ±SD. ***Repeated measures of control group*** Heel vertical velocity = 0.28 ± 0.19 m/s (baseline), 0.30 ± 0.25 m/s (3 weeks later), 0.29 ± 0.24 m/s (7 weeks later). ***Repeated measures of intervention group*** Heel vertical velocity = 0.36 ± 0.27 m/s (baseline), 0.19 ± 0.14 m/s (3 weeks later), 0.29 ± 0.25 m/s (7 weeks later).
D’Angeli, Belvedere, Ortolani, Giannini and Leardini [26]	Data reported as average ± SD Large variations in movement patterns between the 10 points along the tibial shaft during the walking tasks. Peak tibial moments were largest in the medio–lateral axis. Peak tibial moments were comparatively small, and very small in anterior–posterior axis and the proximal–distal axis, respectively. The peak tibial moments were most affected by walking speed in the medio–lateral axis at the distal endpoint (malleoli, P1: low vs. high, *p* < 0.001; low vs. self-selected, *p* = 0.008; high vs. self-selected, *p* < 0.001), but also at the proximal endpoint (tibial plateau, P10: low vs. high, *p* = 0.046; high vs. self-selected, *p* < 0.001). Effects were also observed in the anterior–posterior axis at the distal endpoint between all walking speeds (ankle mortise, P1: low vs. high, *p* < 0.001; low vs. self-selected, *p* = 0.021; high vs. self-selected, *p* < 0.001). ***Author analyses of data reported (see also Figure 2 and Figure 3)*** Peak Tibial Moments were highest in the medio–lateral axis (e.g., self-selected speed = 4.42 ± 1.1 BW.Ht%), followed by the anterior–posterior axis (e.g., self-selected speed = 1.61 ± 0.9 BW.Ht%), and lowest for the proximal–distal axis (e.g., self-selected speed = 0.50 ± 0.2 BW.Ht%) when walking. Peak Tibial Moments were higher at the distal region (e.g., self-selected speed; P1–3 = 7.50 ± 0.9 BW.Ht%) and lower at the mid (e.g., self-selected speed; P4–7 = 4.35 ± 1.0 BW.Ht%) and proximal (e.g., self-selected speed; P8–10 = 1.43 ± 1.2 BW.Ht%) regions in the medio–lateral axis when walking. Variability in the peak tibial moments were generally high (>10%), but appeared highest when the participants were asked to walk slower than their preferred pace, especially in the anterior–posterior axis. The highest variability identified was at the proximal endpoint (tibial plateau: P10) in the medio–lateral axis (127–280%).	Data reported as average ± SD. The GRFs were generally largest in the proximal-distal axis, and increased with speed. The maximum value measured was 1.16 BW for the high-speed walking, followed by 1.10 BW and 1.05 BW for the self-selected and low-speed walking tasks, respectively. All peak GRFs in that axis were in the positive, proximal direction. The GRFs were smallest in the medio–lateral axis, with the peak occurring in the negative, medial direction when walking at low speed (−0.059 BW) but in the positive, lateral direction when walking at self-selected (0.062 BW) and high speed (0.066 BW). All anterior–posterior peak GRFs were in the negative, posterior direction for all walking speeds (slow = −0.361 BW; self-selected = −0.403 BW; high = −0.415 BW). ***Author analyses of data reported*** The variability of the peak GRFs were high (>10%) in the medio–lateral (31%) and proximal–distal (33%) axes for the low walking speed.	
Encarnación-Martínez, Catalá-Vilaplana, Berenguer-Vidal, Sanchis-Sanchis, Ochoa-Puig and Pérez-Soriano [27]	Data reported as average ± SD No significant effect of treadmill or speed for tibial rate (g/ms). ***Curved non-motorised treadmill*** Tibial magnitude was 4.88 ± 1.28, 4.90 ± 1.24 and 5.90 ± 1.44 g at self-selected, 2.77, and 3.33 m/s, respectively. Tibial peak was 4.29 ± 1.05, 4.34 ± 1.06 and 5.06 ± 1.25 g at self-selected, 2.77, and 3.33 m/s, respectively. ***Motorised treadmill*** Tibial magnitude was 5.56 ± 1.18, 5.81 ± 1.06 and 7.10 ± 1.12 g at self-selected, 2.77, and 3.33 m/s, respectively, Tibial peak was 4.78 ± 0.85, 4.97 ± 0.83 and 5.84 ± 1.01 g at self-selected, 2.77, and 3.33 m/s, respectively.	Data reported as average ± SD. No significant effect of treadmill or speed for head peak, head magnitude, or shock attenuation (tibia → head). ***Curved non-motorised treadmill*** Head rate was 59.57 ± 15.27, 58.09 ± 15.98 and 60.61 ± 18.07 g/ms at self-selected, 2.77, and 3.33 m/s, respectively. ***Motorised treadmill*** Head rate was 73.62 ± 15.94, 75.49 ± 19.36 and 80.12 ± 21.2 g/ms at self-selected, 2.77, and 3.33 m/s, respectively.	Data reported as average ± SD. No significant effect of treadmill or speed for stride length or stride frequency. ***Curved non-motorised treadmill*** RPE was 10.46 ± 1.65, 10.54 ± 2.08 and 13.42 ± 2.79 at self-selected, 2.77, and 3.33 m/s, respectively. Heart rate was 156.82 ± 17.01, 158.87 ± 15.81 and 167.55 ± 17.93 bpm at self-selected, 2.77, and 3.33 m/s, respectively. ***Motorised treadmill*** RPE was 9.63 ± 1.90, 9.81 ± 2.27 and 12.44 ± 2.44 at self-selected, 2.77, and 3.33 m/s, respectively. Heart rate was 149.74 ± 17.35, 150.69 ± 17.11 and 161.45 ± 16.77 bpm at self-selected, 2.77, and 3.33 m/s, respectively.
Johnson, Outerleys, Jamison, Tenforde, Ruder and Davis [28]	Data reported as average ± SD (95% confidence interval). Peak vertical tibial acceleration was 8.96 ± 3.09(8.52–9.40), 11.71 ± 3.66(11.19–12.23), 10.92 ± 3.51(10.42–11.42), and 9.94 ± 3.56(9.44–10.45) for the treadmill, marathon 12 km, marathon 23 km, and marathon 40 km assessments, respectively. Peak resultant tibial acceleration was 11.34 ± 3.91(10.78–11.89), 15.72 ± 4.99(15.01–16.43), 14.76 ± 4.85(14.07–15.45), and 13.61 ± 4.89(12.91–14.31) for the treadmill, marathon 12 km, marathon 23 km, and marathon 40 km assessments, respectively. Adjusted tibial acceleration results for speed effects are available in the Supplementary Document, but are not reported here (see Table 1 limitations).		Mean qualifying time was 3:25 (00:21) hrs:min. Rearfoot, midfoot and forefoot strike patterns were 74.0, 15.6, and 10.4%, respectively.
Johnson, Outerleys and Davis [29]	Data reported as median [IQR, Range]. Anterior tibial acceleration peak was 6.44 [5.94, 2.80–13.56] g. Medial tibial acceleration peak was 4.10 [4.01, 2.60–15.71] g. Lateral tibial acceleration peak was 3.96 [4.35, 1.02–9.87] g.	Data reported as median [IQR, Range]. Instantaneous loading rate (ILR). Posterior ILR was 8.56 [6.20, 4.61–17.40] BW/s. Medial ILR was 6.26 [5.99, 3.74–12.07] BW/s. Lateral ILR was 7.48 [11.82, 2.47–20.61] BW/s.	
Meardon, Derrick, Willson, Baggaley, Steinbaker, Marshall and al [30]	Data from Figures 3, 5 and 6 extracted via WebPlot and normalized using reported body mass; estimated averages reported. Peak tibia force in the axial direction was 5.85/5.69, 9.55/9.90, 9.80/10.01 and 10.31/11.10 BW for walking, slow running, running, and fast running in females/males, respectively. Peak tibia force in the anterior–posterior direction was 2.40/2.07, 8.70/6.27, 8.90/6.89 and 9.10/6.75 BW for walking, slow running, running, and fast running in females/males, respectively. Peak tibia force in the medial–lateral direction was 1.60/0.07, 4.20/1.38, 4.50/1.38 and 4.40/1.41 BW for walking, slow running, running, and fast running in females/males, respectively. Peak tibia moment in the torsional direction was 0.02/0.03, 0.08/0.05, 0.08/0.04 and 0.08/0.05 BW.m for walking, slow running, running, and fast running in females/males, respectively. Peak tibia moment in the anterior–posterior direction was 0.13/0.13, 0.27/0.29, 0.28/0.30 and 0.29/0.32 BW.m for walking, slow running, running, and fast running in females/males, respectively. Peak tibia moment in the medial–lateral direction was 0.06/0.06, 0.11/0.08, 0.12/0.09 and 0.12/0.09 BW.m for walking, slow running, running, and fast running in females/males, respectively.	Data from Figures 3, 5 and 6 extracted via WebPlot; estimated averages reported. Peak tibia compression stress was −74.90/−63.85, −152.60/−133.11, −154.97/−138.08, and −161.24/−147.61 MPa for walking, slow running, running, and fast running in females/males, respectively. Peak tibia tension stress was 66.09/54.40, 137.94/115.211, 140.31/120.83, and 143.98/125.79 MPa for walking, slow running, running, and fast running in females/males, respectively. Peak tibia shear stress was 13.78/17.03, 38.22/23.30, 38.01/22.41, and 43.63/26.75 MPa for walking, slow running, running, and fast running in females/males, respectively. Peak tibia compression impulse was −24.35/−21.55, −22.45/−19.21, −21.84/−18.92, and −21.33/−18.96 MPa/s for walking, slow running, running, and fast running in females/males, respectively. Peak tibia tension impulse was 20.73/18.36, 20.77/17.42, 20.37/17.24, and 19.76/17.06 MPa/s for walking, slow running, running, and fast running in females/males, respectively. Peak tibia shear impulse was 0.39/0.30, 0.58/0.24, 0.52/0.20, and 0.58/0.26 MPa/s for walking, slow running, running, and fast running in females/males, respectively.	Data reported as average (95% confidence interval). Preferred running speed: males = 2.97 [2.89–3.05] m/s, females = 2.79 [2.66–2.92] m/s. Weekly training volume: males = 23.34 [17.90–3078] km, females = 30.98 [23.72–38.24].
Mercer, Vance, Hreljac and Hamill [31]	Data reported as average ± SD Peak tibial acceleration was 6.1 ± 1.9, 6.1 ± 1.9, 7.2 ± 1.7, 7.9 ± 1.7, 10.0 ± 2.7, and 10.9 ± 2.8 g for 50, 60, 70, 80, 90, and 100% of maximal running speed, respectively.	Data reported as average ± SD. Peak forehead acceleration was 1.4 ± 0.5, 1.5 ± 0.5, 1.6 ± 0.4, 1.7 ± 0.3, 1.9 ± 0.3, and 1.9 ± 0.3 g for 50, 60, 70, 80, 90, and 100% of maximal running speed, respectively. Shock attenuation was 0.15 ± 0.05, 0.14 ± 0.05, 0.10 ± 0.05, 0.09 ± 0.04, 0.08 ± 0.04, and 0.06 ± 0.03 for 50, 60, 70, 80, 90, and 100% of maximal running speed, respectively.	Data reported as average ± SD. Treadmill running speed was 3.2 ± 0.3, 3.8 ± 0.3, 4.5 ± 0.4, 5.1 ± 0.4, 5.7 ± 0.5, and 6.4 ± 0.5 m/s at 50, 60, 70, 80, 90 and 100% of maximal speed, respectively. Stride length was 2.40 ± 0.17, 2.75 ± 0.22, 3.09 ± 0.23, 3.35 ± 0.25, 3.50 ± 0.22, and 3.68 ± 0.18 m at 50, 60, 70, 80, 90 and 100% of maximal speed, respectively. Stride frequency was 1.33 ± 0.06, 1.37 ± 0.09, 1.45 ± 0.10, 1.52 ± 0.13, 1.63 ± 0.12, and 1.73 ± 0.14 Hz at 50, 60, 70, 80, 90 and 100% of maximal speed, respectively.
Milgrom, Voloshin, Novack, Milgrom, Ekenman and Finestone [32]	Data from table extracted; median [IQR] reported. Peak axial compression was −493.00[178.80] and −927.50[233.95] με at the proximal and distal gauges, respectively when treadmill walking at 1.39 m/s. Peak axial compression was −268.50[83.45] and −1364.70[215.65] με at the proximal and distal gauges, respectively, when treadmill running at 3.61 m/s. Peak axial tension was 187.00[126.40] and −532.50[166.70] με at the proximal and distal gauges, respectively, when treadmill walking at 1.39 m/s. Peak axial tension was 791.80[232.10] and 778.60[50.00] με at the proximal and distal gauges, respectively, when treadmill running at 3.61 m/s. Proximal and distal strain significantly different (*p* ≤ 0.002) for all participants (cases) examined except for Participant 3 (compression, walking) and Participant 2 (tension, running).		
Rice et al. (2024) [33]	Data reported as average ± SD. Resultant bending moment of the distal third of the tibia was 121.78 ± 52.36, 130.38 ± 56.33 and 139.46 ± 59.05 Nm during treadmill running at 2.5, 3.0 and 3.5 m/s, respectively. Resultant bending moments of the tibia generally decreased when running downhill, and increased when running uphill. Peak posterior tibial stress was 72.53 ± 28.23, 77.82 ± 30.31 and 82.69 ± 31.80 MPa during treadmill running at 2.5, 3.0 and 3.5 m/s, respectively. Peak posterior tibial stress generally decreased when running downhill, and increased when running uphill. Peak anterior tibial stress was 53.76 ± 25.70, 57.70 ± 27.71 and 61.96 ± 29.02 MPa during treadmill running at 2.5, 3.0 and 3.5 m/s, respectively. Peak anterior tibial stress generally decreased when running downhill, and increased when running uphill.		Data reported as average ± SD. Ground contact time was 0.27 ± 0.02, 0.25 ± 0.02 and 0.23 ± 0.02 s during treadmill running at 2.5, 3.0 and 3.5 m/s, respectively. Ground contact time did not change with gradient, except for uphill running (+5% = 0.28 ± 0.02 s, +10% = 0.28 ± 0.02 s) at 2.5 m/s. Step frequency was 157.3 ± 7.5, 161.4 ± 8.0 and 166.6 ± 8.6 steps/min during treadmill running at 2.5, 3.0 and 3.5 m/s, respectively. Step frequency generally increased when running uphill and decreased when running downhill.
Rice, Seynnes and Werkhausen [34]	Data from figures extracted via WePlot; estimated averages reported. The peak bending moment of the distal third of the tibia occurred at approximately 62% of stance for both treadmill running speeds. When the weighted vest was worn, the timing of the peak moment was delayed to approximately 66% of stance. The peak bending moment of the distal third of the tibia was 136 and 147 Nm when running at preferred and faster-than-preferred speeds. This increased to 145 and 156 Nm when the weight vest was worn. The cumulative-weighted tibial impulse per kilometre was 224 and 235 Nm.s/km when running at preferred and faster-than-preferred running speeds. It increased to 243 and 255 Nm.s/km when the weight vest was worn.		Data reported as average ± SD. Treadmill running speeds were 3.1 ± 0.3 (preferred) and 3.8 ± 0.4 (preferred + 20%) m/s. Ground contact time was 0.23 ± 0.02 and 0.21 ± 0.02 s when running at preferred and faster-than-preferred speeds. Ground contact time increased when the weight vest was worn to 0.26 ± 0.02 and 0.23 ± 0.02 s. Stride frequency was 2.9 ± 0.2 and 3.1 ± 0.2 Hz when running at preferred and faster-than-preferred speeds. Stride frequency marginally increased when the weight vest was worn to 3.0 ± 0.2 and 3.2 ± 0.2 Hz.
Sheerin, Besier and Reid [35]	Data reported as average ± SD[range]. Resultant peak tibial acceleration (distal) was 9.8 ± 2.7[4.5–19.0], 11.0 ± 3.0[4.9–19.8], 12.1 ± 3.1[5.6–19.7], and 13.5 ± 3.1[6.5–20.6] g when running on the treadmill at 2.7, 3.0, 3.3 and 3.7 m/s, respectively. Symmetry between limbs for resultant peak tibial acceleration was 0.22 ± 5.23, 0.28 ± 5.14, 0.51 ± 4.97, and 0.42 ± 5.17% when running on the treadmill at 2.7, 3.0, 3.3 and 3.7 m/s, respectively, where 0% indicates perfect symmetry between limbs.		Data reported as average ± SD. High-impact runners in the 85 runners increased with treadmill running speed from 20, 22, 21, and 25% when running at 2.7, 3.0, 3.3 and 3.7 m/s. respectively. These runners had a resultant peak tibial acceleration result that was one SD or greater than the group average [48].
Voloshin [36]	Data reported as average ± SD The peak axial acceleration of the proximal tibia (tibial tuberosity) was 2.26 ± 0.05, 2.95 ± 0.25, 4.05 ± 0.17, 4.72 ± 0.26, and 5.65 ± 0.24 g when walking on the treadmill at 0.89, 1.12, 1.34, 1.57, and 1.79 m/s, respectively.		Data reported as average ± SD. Cadence was 50.9 ± 5.1, 54.0 ± 3.2, 57.3 ± 3.1, 61.3 ± 3.5, and 66.5 ± 8.3 steps/min when walking on the treadmill at 0.89, 1.12, 1.34, 1.57, and 1.79 m/s, respectively.

## Data Availability

No new data were created or analysed in this study. Data sharing is not applicable to this article.

## Supplementary Materials

The following supporting information can be downloaded at: https://www.mdpi.com/article/10.3390/s26144401/s1, Table S1: PRISMA-ScR Checklist; Table S2: Checklist that outlines new recommendations for wearable tibial-sensor methodological reporting standards in gait-related biomechanics studies of inertial load and/or shock [62,63,64].

## Abbreviations

The following abbreviations are used in this manuscript:

3D	Three-dimension or three-dimensional
3DMA	Three-dimensional motion analysis
A	Axial (longitudinal) sensor axis
BMI	Body mass index
BW	Body weight
BW/s	Body weight per second
bpm	Beats per minute
cm	Centimetre
FP	Force plate or force platform
g	Gravitational constant unit
gm	Gram
GRF	Ground reaction force
Hr	Hour
Ht	Height
Hz	Hertz
ICC	Intraclass Correlation Coefficient
ILR	Instantaneous loading rate
IMU	Inertial measurement unit
IQR	Interquartile range
kg	Kilogram
Kg/m^2^	Kilogram per metre squared
m	Metre
Min	Minute
MPa	Mega pascal
m/s	Metre per second
MSS	Musculoskeletal
n	Number
Nm	Newton metre
NR	Not reported
P	Position or point
PCC	Population, concept, context
PRISMA-ScR	Preferred reporting items for systematic reviews and meta-analyses extension for scoping reviews
PTA	Peak tibia acceleration
R	Resultant sensor axis
S	Sagittal sensor axis
SD	Standard deviation
SPSS	Statistical Package for the Social Sciences
T	Transverse sensor axis
με	Microstrain where 1 με = 1 × 10^−6^
α	Cronbach’s alpha coefficient
×	Multiply or multiplied
