# Supplementary material for: Non-Standardized Methods of Assessing Tibial Loads During Different Gait Speeds Obscures Load-Management Recommendations in Healthy Adults
Source: Sensors (Basel). 2026 Jul 10;26(14):4401. doi: 10.3390/s26144401 (PMC13417389; doi:10.3390/s26144401)
Supplement: Supplementary file 1 [file sensors-26-04401-s001.zip › sensors-4163617-supplementary.pdf]

Supplementary Table 1. PRISMA-ScR Checklist

Section	Item (No.)	Checklist Item Description	Reported on Page (No.)
**Title**			
Title	1	Identify the report as a scoping review	1
**Abstract**			
Structured summary	2	Provide a structured summary that includes (as applicable) background, objectives, eligibility criteria, sources of evidence, charting methods, results, and conclusions that relate to the review questions and objectives.	1
**Introduction**			
Rationale	3	Describe the rationale for the review in the context of what is already known. Explain why the review questions/objectives lend themselves to a scoping review approach.	2-3
Objectives	4	Provide an explicit statement of the questions and objectives being addressed with reference to their key elements (e.g., population or participants, concepts, and context) or other relevant key elements used to conceptualize the review questions and/or objectives.	3
**Methods**			
Protocol and registration	5	Indicate whether a review protocol exists; state if and where it can be accessed (e.g., a Web address) and if available, provide registration information, including registration number.	3
Eligibility criteria	6	Specify characteristics of the sources of evidence used as eligibility criteria (e.g., years considered, language, and publication status), and provide a rationale.	3
Information sources	7	Describe all information sources in the search (e.g., databases with dates of coverage and contact with authors to identify additional sources), as well as the date the most recent search was executed.	3
Search	8	Present the full electronic search strategy for at least 1 database, including any limits used, such that it could be repeated.	3
Selection of sources of evidence	9	State the process for selecting sources of evidence (i.e., screening and eligibility) included in the scoping review.	3-4

Data charting process	10	Describe the methods of charting data from the included sources of evidence (e.g., calibrated forms or forms that have been tested by the team before their use, and whether data charting was done independently or in duplicate) and any processes for obtaining and confirming data from investigators.	4
Data items	11	List and define all variables for which data were sought and any assumptions and simplifications made.	4
Critical appraisal of individual sources of evidence	12	Optional for scoping reviews. If done, provide a rationale for conducting a critical appraisal of included sources of evidence; describe the methods used and how this information was used in any data synthesis (if appropriate).	4
Summary measures	13	Not applicable for scoping reviews.	
Synthesis of results	14	Describe the methods of handling and summarizing the data that were charted.	4
Risk of bias across studies	15	Not applicable for scoping reviews.	
Additional analyses	16	Not applicable for scoping reviews.	
**Results**			
Selection of sources of evidence	17	Give numbers of sources of evidence screened, assessed for eligibility, and included in the review, with reasons for exclusions at each stage, ideally using a slow diagram.	4-5<br>Figure 1
Characteristics of sources of evidence	18	For each source of evidence, present characteristics for which data were charted and provide the citations.	5<br>Tables 1-2<br>Figure 2
Critical appraisal within sources of evidence	19	If done, present data on critical appraisal of included sources of evidence (see item 12).	Table 1
Results of individual sources of evidence	20	For each included source of evidence, present characteristics for which data were charted and provide the citations.	Tables 1-3<br>Figures 2-4
Synthesis of results	21	Summarize and/or present the charting results as they relate to the review questions and objectives.	5, 29-33
Risk of bias across studies	22	Not applicable for scoping reviews.	
Additional analyses	23	Not applicable for scoping reviews.	
**Discussion**			

Summary of evidence	24	Summarize the main results (including an overview of concepts, themes, and types of evidence available), link to the review questions and objectives, and consider the relevance to key groups.	33-37
Limitations	25	Discuss the limitations of the scoping review process.	37
Conclusions	26	Provide a general interpretation of the results with respect to the review questions and objectives, as well as potential implications and/or next steps.	37-38 Supplemntary Table 2
**Funding**			
Funding sources	27	Describe the sources of funding for the included sources of evidence, as well as sources of funding for the scoping review. Describe the role of the funders of the scoping review.	38

Supplementary Table 2. Checklist that outlines new recommendations for wearable tibial sensor methodological reporting standards in gait-related biomechanics studies of inertial load and/or shock

**Wearable Sensor**		
1	Wearable sensor and software system including version, model, company, and origin	
2	Sensor mass and dimensions	
3	Sensor sampling frequency and maximum measurement thresholds	
4	Skin preparation, if any, prior to sensor placement	
5	Sensor positions stated with respect to:<br>• bony landmarks<br>• preferably scaled to the participant's anthropometry (e.g., as a percentage of limb length) instead of absolute (e.g., 3 cm above bony landmark)<br>• alignment of sensor axes<br>• alignment of segment and/or bone axes	
6	Method of sensor fixation including type (e.g., rigid, semi-elastic, elastic), model, company, and origin of any consumables used such as adhesive tape	
**Gait Assessment Protocol**		
7	Description of the testing environment (Indoor, Outdoor) and ground surface	
8	If using a treadmill, details on whether it was motorized or non-motorized, the belt length and width, model, company, and origin, and the gradient set (e.g., 0° level, 2° incline, -2° decline)	
9	If overground, include the length of the overall walkway/runway, the data collection zone, the length pre- and post-data collection zone, and the position of force platforms (if relevant)	

10	Report on whether the gait speed was self-selected or set (imposed), how it was monitored, and whether there were any criteria for task consistency/minimization of variability within trial and/or between trials	
11	Report on the footwear worn, whether provided or the participant's preferred footwear. If there was more than one testing session, whether the participant was instructed to wear the same footwear to all sessions	
12	If studying running, report on the foot-strike pattern for all participants	
13	It is recommended to also report on the preferred limb/footedness) of the participants using a validated questionnaire [e.g., 62, 63]	
**Data Analyses**		
14	Data processing procedures (e.g., smoothing and filtering), measurement modality (e.g., PTA-A, PTA-R) and identification of points/events of interest, including gait cycle/event definitions and justification of methods selected [64]	
15	Normalization of measures, where appropriate, consistent with standard conventions in published literature (e.g., Linear acceleration; 1 g = 9.81 m/s^2)	
16	Reliability and validity of methods clearly reported, preferably from in-house testing [64]	
